# Supplementary material for: Stimulus-selective crosstalk via the NF-κB signaling system reinforces innate immune response to alleviate gut infection
Source: eLife. 2015 Apr 23;4:e05648. doi: 10.7554/eLife.05648 (PMC4432492; doi:10.7554/eLife.05648)
Supplement: Figure 3—source data 1. — DOI: http://dx.doi.org/10.7554/eLife.05648.010 [file elife05648s001.doc]

**Figure 3- source data 1: List of** **LPS target genes positively regulated through crosstalk**

| **Name** | **Crosstalk Score** | **Name** | **Crosstalk Score** |
| --- | --- | --- | --- |
| Csf2 | 5.969268 | Reep3 | 0.2418609 |
| Il1b | 5.554102 | Ass1 | 0.23933193 |
| Il1a | 3.989716 | S100a8 | 0.2370591 |
| H2-M2 | 1.931003 | Rs1 | 0.23173966 |
| Il6 | 1.3107078 | Gmfg | 0.22012536 |
| Ccl5 | 1.0115684 | Fermt3 | 0.21617046 |
| Mmp9 | 0.8766486 | Ch25h | 0.20735942 |
| Susd2 | 0.8371903 | Grem2 | 0.20216912 |
| Stac2 | 0.8271197 | Slc7a11 | 0.2008302 |
| Bcl2a1c | 0.77956426 | Asah3l | 0.19902064 |
| Slamf8 | 0.77935827 | A330021E22Rik | 0.19773032 |
| 2010005H15Rik | 0.7419086 | Creb5 | 0.18704239 |
| Dner | 0.66452736 | Crybg3 | 0.18583636 |
| Ccl3 | 0.57391673 | Cd14 | 0.18038304 |
| AA691260 | 0.5665377 | Mcoln2 | 0.1704542 |
| Tnfaip3 | 0.559966 | D12Ertd553e | 0.16052525 |
| Cd74 | 0.54834116 | Tm4sf1 | 0.15555795 |
| Areg | 0.53293806 | D9Wsu20e | 0.15543577 |
| Islr | 0.4873252 | Acsl1 | 0.15292066 |
| Indo | 0.48592317 | Trps1 | 0.15163304 |
| Acot4 | 0.48040023 | Zfpm2 | 0.14772816 |
| Bckdhb | 0.46314272 | Sc5d | 0.14517863 |
| Slc6a12 | 0.43394518 | Trp53inp2 | 0.1450395 |
| Ppp1r12c | 0.4259527 | Hcls1 | 0.13905674 |
| EG433016 | 0.38811126 | Unc5b | 0.13403098 |
| Sema4a | 0.37136233 | Rbp1 | 0.1263234 |
| OTTMUSG00000000971 | 0.35686472 | Gprc5b | 0.12606286 |
| Rhof | 0.3464215 | Pla1a | 0.12505278 |
| Slc2a6 | 0.3402488 | Prkcb | 0.11522357 |
| 1110048D14Rik | 0.33979535 | Icam1 | 0.11317971 |
| Selp | 0.33039758 | Hapln4 | 0.10988372 |
| Zkscan3 | 0.31930265 | Cxxc4 | 0.10834137 |
| Ngfb | 0.31811228 | En1 | 0.097422175 |
| Upp1 | 0.30201074 | Tnip1 | 0.09580752 |
| Rasa2 | 0.28887904 | LOC100044177 | 0.09524912 |
| Ccl4 | 0.28617856 | Clcn3 | 0.094447844 |
| Igfbp5 | 0.2851165 | Meox2 | 0.08661236 |
| Emr1 | 0.27243835 | LOC100048710 | 0.0837287 |
| Cd40 | 0.2519999 | Per1 | 0.08001423 |

| **Name** | **Crosstalk Score** |
| --- | --- |
| Adm | 0.07977864 |
| Tnfaip8 | 0.07946285 |
| Pilrb1 | 0.07577487 |
| Arhgap22 | 0.07411319 |
| BC062115 | 0.07226331 |
| Gadd45a | 0.07224684 |
| 9530096D07Rik | 0.07192086 |
| Zswim3 | 0.069966614 |
| Cxcl1 | 0.06906108 |
| Col5a3 | 0.06777434 |
| Col3a1 | 0.066022255 |
| Smpdl3a | 0.059856854 |
| Adam17 | 0.05642737 |
| Trib2 | 0.05392169 |
| 9030425E11Rik | 0.05207676 |
| Lrig1 | 0.04716315 |
| Slc11a1 | 0.042424396 |
| Aldh1a2 | 0.03944287 |
| Camk2n1 | 0.03731716 |
| 2310016C08Rik | 0.036710996 |
| Ccl2 | 0.031668577 |
| Prr16 | 0.029613456 |
| Tspan7 | 0.028081112 |
| Klhl10 | 0.027619595 |
| Trfr2 | 0.026717912 |
| Rassf4 | 0.023559801 |
| Gprc5a | 0.020169225 |
| Tnfaip2 | 0.019850155 |
| Pik3c2a | 0.013207489 |
| Rffl | 0.012740468 |
| Hmox1 | 0.012432995 |
| Cd59b | 0.011475573 |
| Rnaset2 | 0.010810015 |
| Eif3h | 0.010757623 |
| Ly9 | 0.010371544 |
| Hsd17b11 | 0.001481471 |
