# Supplementary material for: Stimulus-selective crosstalk via the NF-κB signaling system reinforces innate immune response to alleviate gut infection
Source: eLife. 2015 Apr 23;4:e05648. doi: 10.7554/eLife.05648 (PMC4432492; doi:10.7554/eLife.05648)
Supplement: Figure 3—source data 2. — DOI: http://dx.doi.org/10.7554/eLife.05648.011 [file elife05648s002.doc]

**Figure 3- source data 2:** **a pre-determined list of 290 NF-κB response genes used in GSEA**

| 1110038F14Rik | Hnrpa0 | Rgs16 | Wnt10b | Edf1 |
| --- | --- | --- | --- | --- |
| 2810474O19Rik | Ibrdc3 | Rin3 | Zbtb7c | EG432466 |
| 3110043O21Rik | Icam1 | Ripk2 | Zfp429 | Egln3 |
| 4631426J05Rik | Irf1 | Rnd1 | Zswim4 | Eif6 |
| Akap2 | Klhl25 | Rnf125 | 1190003J15Rik | Enpp2 |
| Arhgap22 | LOC100047353 | Rrad | 1200009I06Rik | Fas |
| Arrdc3 | LOC100047934 | Sele | 2310016C08Rik | Fbxl5 |
| Bahcc1 | Map3k14 | Selp | 4930519N13Rik | Fcgr2b |
| BC016423 | Micall2 | Sema4b | A030007L17Rik | Flrt2 |
| Bcl3 | Mtmr14 | Slc7a7 | A430085C19 | Gadd45a |
| Bcor | Nfatc1 | Slco4a1 | Acpp | Gch1 |
| Bhlhb5 | Nfkb1 | Snf1lk | Adamts4 | Gpr84 |
| Birc2 | Nfkb2 | Spata13 | Adarb1 | Gsta3 |
| Ccl1 | Nfkbia | Sqstm1 | Aif1 | H2-Q7 |
| Ccl2 | Nfkbib | Stat5a | Angptl4 | Hcls1 |
| Ccl7 | Nfkbie | Stk17b | Arhgef3 | Htra2 |
| Cdc37 | Nr6a1 | Stx11 | Arl6ip5 | Hvcn1 |
| Cdkn1a | Optn | Tgfb1 | Ass1 | Ifnar2 |
| Cebpb | Pard6g | Tjp2 | Batf | Igfbp3 |
| Ch25h | Pdgfb | Tlr2 | C3 | Il15ra |
| Csf1 | Pfkfb3 | Tmc7 | Ccl5 | Il1rl1 |
| Cxcl1 | Pigh | Tnfaip2 | Ccl9 | Il2rg |
| Cyp26b1 | Plekhg2 | Tnfaip3 | Cdc42ep5 | Il4i1 |
| Ehd1 | Plxna2 | Tnfsf11 | Chic2 | Lilrb4 |
| Fbxo32 | Pnkd | Top1 | Clec4d | Lincr |
| Foxs1 | Pnpla3 | Trim47 | Clec4n | LOC100044702 |
| Fscn1 | Ralgds | Uap1 | Col18a1 | LOC100045040 |
| Gdpd5 | Rbm47 | Ubtd2 | Cx3cl1 | LOC100048710 |
| Gfpt2 | Rela | Vcam1 | Cxcl16 | LOC665181 |
| Gjb3 | Relb | Vgll4 | Cyp7b1 | Lrig1 |

| Map3k6 | S100a8 | Tnip1 | Erdr1 | Rasa2 |
| --- | --- | --- | --- | --- |
| Mapk11 | Saa3 | Traf1 | Ets2 | Rasgef1b |
| Mast4 | Samsn1 | Traf3 | Fpr2 | Rassf4 |
| Mcpt8 | Sbno2 | Tslp | Gdnf | Rgs1 |
| Mina | Scin | Twist2 | Gpr109a | Rnf14 |
| Mmp13 | Sema4a | Tyk2 | Gpr18 | Sdc1 |
| Mmp3 | Serpina3f | Ubd | Hmox1 | Sdc4 |
| Mmp9 | Serpina3g | A330021E22Rik | Ifitm5 | Sgms2 |
| Mrpl38 | Serpina3n | AA960436 | Il10 | Slc39a14 |
| Mt2 | Slc11a2 | Ace | Il10ra | Socs3 |
| Nfe2l2 | Slc25a37 | Apol7c | Il1a | Srgn |
| Ninj1 | Slc2a6 | Arl5c | Il1b | Stap1 |
| P2ry2 | Slc39a4 | Bcl2a1a | Il27 | Tank |
| Pdlim4 | Slc43a3 | Bcl2a1b | Il6 | Tmem2 |
| Plscr1 | Slc7a2 | Btg1 | Itgal | Tnf |
| Prg4 | Smox | Ccl17 | Lad1 | Tnfrsf1b |
| Psmd10 | Smpdl3b | Ccl3 | Lcp2 | Tnfsf15 |
| Ptger2 | Sod2 | Ccl4 | LOC100048553 | Zc3h12a |
| Ptges | Sod3 | Ccrl2 | Mapkapk2 | Zeb2 |
| Ptgir | Srxn1 | Cd14 | Marcksl1 | Zfp263 |
| Ptx3 | St3gal1 | Cd40 | Mtmr14 |  |
| Rab20 | Steap4 | Cd69 | Mycl1 |  |
| Rab32 | Stk10 | Clec4e | Myo1g |  |
| Rab8b | Stx6 | Csf2 | Ndrg2 |  |
| Rbpj | Tacc1 | Csf3 | Nts |  |
| Rbpms | Tagln2 | Cxcl2 | Nupr1 |  |
| Rcl1 | Tank | Denr | Pim1 |  |
| Rcsd1 | Tmem132e | Dusp2 | Plagl2 |  |
| Rhbdf2 | Tmem51 | Ebi3 | Plek |  |
| Rttn | Tnfrsf22 | Edn1 | Ppp1r15b |  |

The list of RelA NF-κB target genes, obtained from Alexander Hoffmann, UCLA was arrived onto using genetics and systems biology tools.
