# Supplementary material for: Stimulus-selective crosstalk via the NF-κB signaling system reinforces innate immune response to alleviate gut infection
Source: eLife. 2015 Apr 23;4:e05648. doi: 10.7554/eLife.05648 (PMC4432492; doi:10.7554/eLife.05648)
Supplement: Supplementary file 1. — List of biochemical species and their initial concentrations in the model. DOI: http://dx.doi.org/10.7554/eLife.05648.020 [file elife05648s003.docx]

**Supplementary file 1. List of biochemical species and their initial concentrations in the model**

| **Model Species** |  |  | **Nomenclature** |  | **Initial**  **amount(in nM)** | | **Location** |
| --- | --- | --- | --- | --- | --- | --- | --- |
| IkBα |  |  | IkBa |  | 0 |  | cytoplasm |
| IkBαn |  |  | IkBan |  | 0 |  | nucleus |
| IkBα mRNA |  |  | tIkBa |  | 0 |  | cytoplasm |
| IkBβ |  |  | IkBb |  | 0 |  | cytoplasm |
| IkBβn |  |  | IkBbn |  | 0 |  | nucleus |
| IkBβ mRNA |  |  | tIkBb |  | 0 |  | cytoplasm |
| IkBε |  |  | IkBe |  | 0 |  | cytoplasm |
| IkBεn |  |  | IkBen |  | 0 |  | nucleus |
| IkBε mRNA |  |  | tIkBe |  | 0 |  | cytoplasm |
| IkBδ |  |  | IkBd |  | 0 |  | cytoplasm |
| IkBδn |  |  | IkBdn |  | 0 |  | nucleus |
| p100 |  |  | p100 |  | 0 |  | cytoplasm |
| NFkB2 mRNA |  |  | tp100 |  | 0 |  | cytoplasm |
| RelA:p50 |  |  | RelA:p50 |  | 0 |  | cytoplasm |
| RelA:p50n |  |  | RelA:p50n |  | 0 |  | nucleus |
| NFkB1 mRNA |  |  | tNFkB1 |  | 0 |  | cytoplasm |
| RelA:p52 |  |  | RelA:p52 |  | 0 |  | cytoplasm |
| RelA:p52n |  |  | RelA:p52n |  | 0 |  | nucleus |
| IkBα:RelA:p50 |  |  | IkBaN:RelA:p50 |  | 0 |  | cytoplasm |
| IkBα:RelA:p50n |  |  | IkBa:RelA:p50n |  | 0 |  | nucleus |
| IkBβ: RelA:p50 |  |  | IkBb:RelA:p50 |  | 0 |  | cytoplasm |
| IkBβ:RelA:p50n |  |  | IkBb:RelA:p50n |  | 0 |  | nucleus |
| IkBε:RelA:p50 |  |  | IkBe:RelA:p50 |  | 0 |  | cytoplasm |
| IkBε:RelA:p50n |  |  | IkBe:RelA:p50n |  | 0 |  | nucleus |
| IkBδ:RelA:p50 |  |  | IkBd:RelA:p50 |  | 0 |  | cytoplasm |
| IkBδ:RelA:p50n |  |  | IkBd:RelA:p50n |  | 0 |  | nucleus |
| IkBα:RelA:p52 |  |  | IkBa:RelA:p52 |  | 0 |  | cytoplasm |
| IkBα:RelA:p52n |  |  | IkBa:RelA:p52n |  | 0 |  | nucleus |
| IkBβ:RelA:p52 |  |  | IkBb:RelA:p52 |  | 0 |  | cytoplasm |
| IkBβ:RelA:p52n |  |  | IkBb:RelA:p52n |  | 0 |  | nucleus |
| IkBε:RelA:p52 |  |  | IkBe:RelA:p52 |  | 0 |  | cytoplasm |
| IkBε:RelA:p52n |  |  | IkBe:RelA:p52n |  | 0 |  | nucleus |
| IkBδ:RelA:p52 |  |  | IkBd:RelA:p52 |  | 0 |  | cytoplasm |
| IkBδ:RelA:p52n |  |  | IkBd:RelA:p52n |  | 0 |  | nucleus |
| NEMO-IKK |  |  | NEMO-IKK |  | 1 |  | cytoplasm |
| NIK-IKK1 |  |  | NIK-IKK1 |  | 1 |  | cytoplasm |
