# Supplementary material for: Stimulus-selective crosstalk via the NF-κB signaling system reinforces innate immune response to alleviate gut infection
Source: eLife. 2015 Apr 23;4:e05648. doi: 10.7554/eLife.05648 (PMC4432492; doi:10.7554/eLife.05648)
Supplement: Supplementary file 2. — List of biochemical reactions and rate constants in the model. DOI: http://dx.doi.org/10.7554/eLife.05648.021 [file elife05648s004.docx]

**Supplementary file 2.** **List of biochemical reactions and rate constants in the model**

| **Param**  **No.** | **Reaction** | | **Parameter**  **Value** | **Category** | **Unit** | | **Location** | | | **Reference** | |
| --- | --- | --- | --- | --- | --- | --- | --- | --- | --- | --- | --- |
| **1** | 🡺tIkBa | | 6.20E-02 | RNA synthesis  (constitutive) | nM  min^-1^ | | ___ | | | refined the published parameter value[^1^](#_ENREF_1) with a 4.8 fold increase based on our experimental measurements | |
| **2** | 🡺tIkBb | | 2.00E-03 | RNA synthesis  (constitutive) | nM  min^-1^ | | ___ | | | fitted by increasing the published parameter[^1^](#_ENREF_1) value by 3 fold | |
| **3** | 🡺tIkBe | | 2.50E-04 | RNA synthesis  (constitutive) | nM  min^-1^ | | ___ | | | fitted by increasing the published parameter value[^1^](#_ENREF_1) by 2 fold | |
| **4** | 🡺tp100 | | 1.90E-04 | RNA synthesis  (constitutive) | nM  min^-1^ | | ___ | | | refined the published parameter value[^1^](#_ENREF_1) with a 6.3 fold increase based on our experimental measurements | |
| **5** | 🡺tNFkB1 | | 1.40E-05 | RNA synthesis  (constitutive) | nM  min^-1^ | | ___ | | | refined the published parameter value[^1^](#_ENREF_1) with a 2 fold decrease to achieve 100-150nM NFκB peak activity in TNF regime | |
| **6** | 🡺tIkBa  (Induced by RelA:p50) | | 4.00E-07 | RNA synthesis  delay 0 min,  Hill coefficient 3 | nM^-2^  min^-1^ | | ___ | | | parameterized to achieve ~5 fold induction of IκBα mRNA upon stimulation based on the experimental data (Figure 5A) | |
| **7** | 🡺tIkBb  (Induced by RelA:p50) | | 1.20E-08 | RNA synthesis  delay 37 min,  Hill coefficient 3 | nM^-2^  min^-1^ | | ___ | | | fitted by increasing the published parameter value[^2^](#_ENREF_2) by 1.7 fold | |
| **8** | 🡺tIkBe  (Induced by RelA:p50) | | 5.00E-09 | RNA synthesis  delay 37min,  Hill coefficient 3 | nM^-2^  min^-1^ | | ___ | | | parameterized to achieve similar fold inductions of IκBα and IkBe mRNAs upon stimulation | |
| **9** | 🡺tp100  (Induced by RelA:p50) | | 2.00E-08 | RNA synthesis  delay 60min,  Hill coefficient 3 | nM^-2^  min^-1^ | | ___ | | | refined the published parameter value[^3^](#_ENREF_3) with a 1.25 fold decrease based on our experimental measurements (Figure 5A and 5B). | |
| **10** | 🡺tIkBa  (Induced by RelA:p52) | | 4.00E-07 | RNA synthesis  delay 0 min,  Hill coefficient 3 | nM^-2^  min^-1^ | | ___ | | | assumed to be identical to that of RelA:p50 dimer (6), based on appendix figure 4B presented in the Appendix-1 | |
| **11** | 🡺tIkBb  (Induced by RelA:p52) | | 1.20E-08 | RNA synthesis  delay 37 min,  Hill coefficient 3 | nM^-2^  min^-1^ | | ___ | | | similarly assumed to be identical to that of RelA:p50 dimer (7), | |
| **12** | 🡺tIkBe  (Induced by RelA:p52) | | 5.00E-09 | RNA synthesis  delay 37 min,  Hill coefficient 3 | nM^-2^  min^-1^ | | ___ | | | similarly assumed to be identical to that of RelA:p50 dimer (8), | |
| **13** | 🡺tp100  (Induced by RelA:p52) | | 2.00E-08 | RNA synthesis  delay 60min,  Hill coefficient 3 | nM^-2^  min^-1^ | | ___ | | | assumed to be identical to that of RelA:p50 dimer (9), based on appendix figure 4C presented in the Appendix-1 | |
| **14** | tIkBa 🡺 | | 3.50E-02 | RNA degradation | min^-1^ | | cytoplasm | | | identical as in[^3^](#_ENREF_3) | |
| **15** | tIkBb 🡺 | | 3.00E-03 | RNA degradation | min^-1^ | | cytoplasm | | | identical as in[^3^](#_ENREF_3) | |
| **16** | tIkBe 🡺 | | 4.00E-03 | RNA degradation | min^-1^ | | cytoplasm | | | identical as in[^3^](#_ENREF_3) | |
| **17** | tp100 🡺 | | 1.60E-03 | RNA degradation | min^-1^ | | cytoplasm | | | parameterized based on the experimental data presented in appendix figure 2B | |
| **18** | tNFkB1 🡺 | | 1.00E-03 | RNA degradation | min^-1^ | | cytoplasm | | | composite species, constrained the parameter value to achieve routinely observed 100-150nM NFκB peak activity in TNF regime | |
| **19** | 🡺IkBa | | 1.00E+00 | protein synthesis | min^-1^ | | cytoplasm | | | constrained considering the published rate for polypeptide synthesis[^4^](#_ENREF_4) and the molecular size of the protein | |
| **20** | 🡺IkBb | | 1.00E+00 | protein synthesis | min^-1^ | | cytoplasm | | | constrained considering the published rate for polypeptide synthesis[^4^](#_ENREF_4) and the molecular size of the protein | |
| **21** | 🡺IkBe | | 1.00E+00 | protein synthesis | min^-1^ | | cytoplasm | | | constrained considering the published rate for polypeptide synthesis[^4^](#_ENREF_4) and the molecular size of the protein | |
| **22** | 🡺p100 | | 5.00E-01 | protein synthesis | min^-1^ | | cytoplasm | | | constrained considering the published rate for polypeptide synthesis[^4^](#_ENREF_4) and the molecular size of the protein | |
| **23** | 🡺RelA:p50 | | 1.00E+00 | protein synthesis | min^-1^ | | cytoplasm | | | composite species, constrained considering the published rate for polypeptide synthesis[^4^](#_ENREF_4) and the molecular size of the protein | |
| **24** | IκBα 🡺 | | 1.38E-01 | constitutive NFkB  or IkB degradation | min^-1^ | | nucleus & cytoplasm | | | refined the published parameter value[^2^](#_ENREF_2) with a 1.1 fold increase based on our measurements (appendix figure 3A) | |
| **25** | IkBb 🡺 | | 2.07E-01 | constitutive NFkB  or IkB degradation | min^-1^ | | nucleus & cytoplasm | | | similarly refined the published parameter value[^2^](#_ENREF_2) with a 1.1 fold increase for fitting | |
| **26** | IkBe 🡺 | | 1.73E-01 | constitutive NFkB  or IkB degradation | min^-1^ | | nucleus & cytoplasm | | | identical to the previously published parameter value[^2^](#_ENREF_2) | |
| **27** | IkBd 🡺 | | 2.40E-04 | constitutive NFkB  or IkB degradation | min^-1^ | | nucleus & cytoplasm | | | fitted based on the experimental data presented in appendix figure 3A. | |
| **28** | p100 🡺 | | 4.00E-01 | constitutive NFkB  or IkB degradation | min^-1^ | | nucleus & cytoplasm | | | free monomer p100 which was assumed to be unstable, similar to free IκBα | |
| **29** | RelA:p50 🡺 | | 2.40E-04 | constitutive NFkB  or IkB degradation | min^-1^ | | nucleus & cytoplasm | | | identical as in[^1^](#_ENREF_1) | |
| **30** | RelA:p52 🡺 | | 2.40E-04 | constitutive NFkB  or IkB degradation | min^-1^ | | nucleus & cytoplasm | | | assumed to be identical to that of RelA:p50 dimer (29), | |
| **31** | IkBa + NEMO-IKK🡺 | | 1.95E-03 | NEMO mediated  free IkB degradation | nM^-1^  min^-1^ | | cytoplasm | | | refined the published parameter value[^1^](#_ENREF_1) with a 1.4 fold increase based on our measurements (appendix figure 3B) suggesting similar degradation of bound and free IκBs. | |
| **32** | IkBb + NEMO-IKK🡺 | | 5.00E-04 | NEMO mediated  free IkB degradation | nM^-1^  min^-1^ | | cytoplasm | | | similarly refined the published parameter value[^1^](#_ENREF_1) with a 1.1 fold increase for fitting | |
| **33** | IkBe + NEMO-IKK🡺 | | 5.00E-04 | NEMO mediated  free IkB degradation | nM^-1^  min^-1^ | | cytoplasm | | | similarly refined the published parameter value[^1^](#_ENREF_1) with a 1.8 fold decrease for fitting | |
| **34** | IkBd+NIK-IKK1 🡺 | | 1.00E-03 | NIK mediated  free IkBd degradation | nM^-1^  min^-1^ | | cytoplasm | | | fitted based on the experimental data presented in appendix figure 3C. | |
| **35** | IkBa 🡺IkBan | | 9.00E-02 | nuclear import | min^-1^ | | ___ | | | identical as in[^2^](#_ENREF_2) |  |
| **36** | IkBb 🡺IkBbn | | 9.00E-03 | nuclear import | min^-1^ | | ___ | | | identical as in[^2^](#_ENREF_2) |  |
| **37** | IkBe 🡺IkBen | | 4.50E-02 | nuclear import | min^-1^ | | ___ | | | identical as in[^2^](#_ENREF_2) |  |
| **38** | IkBd 🡺IkBdn | | 4.50E-02 | nuclear import | min^-1^ | | ___ | | | identical as in[^2^](#_ENREF_2) |  |
| **39** | RelA:p50 🡺 RelA:p50n | | 5.40E+00 | nuclear import | min^-1^ | | ___ | | | identical as in[^2^](#_ENREF_2) |  |
| **40** | RelA:p52 🡺 RelA:p52n | | 5.40E+00 | nuclear import | min^-1^ | | ___ | | | assumed to be similar to that of RelA:p50 dimer (39). Note, nuclear import of NF-κB dimers are largely determined by the nuclear localization signal present in RelA. |  |
| **41** | IkBan 🡺IkBa | | 1.20E-02 | nuclear export | min^-1^ | | ___ | | | identical as in[^2^](#_ENREF_2) |  |
| **42** | IkBbn 🡺IkBb | | 1.20E-02 | nuclear export | min^-1^ | | ___ | | | identical as in[^2^](#_ENREF_2) |  |
| **43** | IkBen 🡺IkBe | | 1.20E-02 | nuclear export | min^-1^ | | ___ | | | identical as in[^2^](#_ENREF_2) |  |
| **44** | IkBdn 🡺IkBd | | 1.20E-02 | nuclear export | min^-1^ | | ___ | | | identical as in[^2^](#_ENREF_2) |  |
| **45** | RelA:p50n 🡺RelA:p50 | | 1.80E-03 | nuclear export | min^-1^ | | ___ | | | fitted by decreasing the published parameter value[^2^](#_ENREF_2) by 2.7 fold |  |
| **46** | RelA:p52n 🡺 RelA:p52 | | 1.80E-03 | nuclear Export | min^-1^ | | ___ | | | assumed to be similar to that of RelA:p50 dimer (45) |  |
| **47** | RelA:p50 + IkBa  🡺 IkBa:RelA:p50 | | 3.00E-02 | NFkB IkB association | nM^-1^  min^-1^ | | cytoplasm & nucleus | | | identical as in[^2^](#_ENREF_2) |  |
| **48** | RelA:p50 + IkBb  🡺 IkBb:RelA:p50 | | 3.00E-02 | NFkB IkB association | nM^-1^  min^-1^ | | cytoplasm & nucleus | | | identical as in[^2^](#_ENREF_2) |  |
| **49** | RelA:p50 + IkBe  🡺 IkBe:RelA:p50 | | 3.00E-02 | NFkB IkB association | nM^-1^  min^-1^ | | cytoplasm & nucleus | | | identical as in[^2^](#_ENREF_2) |  |
| **50** | RelA:p50 + IkBd  🡺 IkBd:RelA:p50 | | 3.00E-02 | NFkB IkB association | nM^-1^  min^-1^ | | cytoplasm & nucleus | | | identical as in[^2^](#_ENREF_2) |  |
| **51** | RelA:p52 + IkBa  🡺IkBa:RelA:p52 | | 1.50E-03 | NFkB IkB association | nM^-1^  min^-1^ | | cytoplasm & nucleus | | | relative to RelA:p50 dimer, RelA:p52 binding to IkBs were weak (appendix figure 4D). Accordingly, slower association rates for RelA:p52 binding to IkBs were used. |  |
| **52** | RelA:p52 + IkBb  🡺IkBb:RelA:p52 | | 1.50E-03 | NFkB IkB association | nM^-1^  min^-1^ | | cytoplasm & nucleus | | | same as 51. |  |
| **53** | RelA:p52 + IkBe  🡺IkBe:RelA:p52 | | 1.50E-03 | NFkB IkB association | nM^-1^  min^-1^ | | cytoplasm & nucleus | | | same as 51. |  |
| **54** | RelA:p52 + IkBd  🡺IkBd:RelA:p52 | | 1.50E-03 | NFkB IkB association | nM^-1^  min^-1^ | | cytoplasm & nucleus | | | same as 51. |  |
| **55** | IkBa:RelA:p50  🡺RelA:p50 + IkBa | | 6.00E-05 | NFkB:IkB complex dissociation | min^-1^ | | cytoplasm &nucleus | | | identical as in[^2^](#_ENREF_2) |  |
| **56** | IkBb:RelA:p50  🡺RelA:p50 + IkBb | | 6.00E-05 | NFkB:IkB complex dissociation | min^-1^ | | cytoplasm &nucleus | | | identical as in[^2^](#_ENREF_2) |  |
| **57** | IkBe:RelA:p50  🡺RelA:p50 + IkBe | | 6.00E-05 | NFkB:IkB complex dissociation | min^-1^ | | cytoplasm &nucleus | | | identical as in[^2^](#_ENREF_2) |  |
| **58** | IkBd:RelA:p50  🡺RelA:p50 + IkBd | | 6.00E-05 | NFkB:IkB complex dissociation | min^-1^ | | cytoplasm &nucleus | | | identical as in[^2^](#_ENREF_2) |  |
| **59** | IkBa:RelA:p52  🡺RelA:p52 + IkBa | | 6.00E-05 | NFkB:IkB complex dissociation | min^-1^ | | cytoplasm &nucleus | | | assumed to be similar to that of RelA:p50 dimer (55). |  |
| **60** | IkBb:RelA:p52  🡺RelA:p52 + IkBb | | 6.00E-05 | NFkB:IkB complex dissociation | min^-1^ | | cytoplasm &nucleus | | | assumed to be similar to that of RelA:p50 dimer (56). |  |
| **61** | IkBe:RelA:p52  🡺RelA:p52 + IkBe | | 6.00E-05 | NFkB:IkB complex dissociation | min^-1^ | | cytoplasm &nucleus | | | assumed to be similar to that of RelA:p50 dimer (57). |  |
| **62** | IkBd:RelA:p52  🡺RelA:p52 + IkBd | | 6.00E-05 | NFkB:IkB complex dissociation | min^-1^ | | cytoplasm & nucleus | | | assumed to be similar to that of RelA:p50 dimer (58). |  |
| **63** | IκBα:RelA:p50  🡺 RelA:p50 | | 6.00E-05 | constitutive degradation of IkB within NFkB:IkB complex | min^-1^ | | cytoplasm &nucleus | | | identical as in[^2^](#_ENREF_2) |  |
| **64** | IkBb: RelA:p50  🡺 RelA:p50 | | 6.00E-05 | constitutive degradation of IkB within NFkB:IkB complex | min^-1^ | | cytoplasm &nucleus | | | identical as in[^2^](#_ENREF_2) |  |
| **65** | IkBe:RelA:p50  🡺 RelA:p50 | | 6.00E-05 | constitutive degradation of IkB within NFkB:IkB complex | min^-1^ | | cytoplasm &nucleus | | | identical as in[^2^](#_ENREF_2) |  |
| **66** | IkBd:RelA:p50  🡺 RelA:p50 | | 6.00E-05 | constitutive degradation of IkB within NFkB:IkB complex | min^-1^ | | cytoplasm &nucleus | | | identical as in[^2^](#_ENREF_2) |  |
| **67** | IκBα:RelA:p52  🡺 RelA:p52 | | 6.00E-05 | constitutive degradation of IkB within NFkB:IkB complex | min^-1^ | | cytoplasm &nucleus | | | assumed to be similar to that of RelA:p50 dimer (63). |  |
| **68** | IkBb:RelA:p52  🡺 RelA:p52 | | 6.00E-05 | constitutive degradation of IkB within NFkB:IkB complex | min^-1^ | | cytoplasm &nucleus | | | assumed to be similar to that of RelA:p50 dimer (64). |  |
| **69** | IkBe:RelA:p52  🡺 RelA:p52 | | 6.00E-05 | constitutive degradation of IkB within NFkB:IkB complex | min^-1^ | | cytoplasm &nucleus | | | assumed to be similar to that of RelA:p50 dimer (65). |  |
| **70** | IkBd: RelA:p52  🡺 RelA:p52 | | 6.00E-05 | constitutive degradation of IkB within NFkB:IkB complex | min^-1^ | | cytoplasm &nucleus | | | assumed to be similar to that of RelA:p50 dimer (66). |  |
| **71** | IkBa: RelA:p50 🡺IkBa | | 6.00E-05 | constitutive degradation of NFkB within NFkB:IkB complex | min^-1^ | | cytoplasm & nucleus | | | fitted by decreasing the published parameter value[^1^](#_ENREF_1) by 4 fold |  |
| **72** | IkBb:RelA:p50 🡺IkBb | | 6.00E-05 | constitutive degradation of NFkB within NFkB:IkB complex | min^-1^ | | cytoplasm & nucleus | | | fitted by decreasing the published parameter value[^1^](#_ENREF_1) by 4 fold |  |
| **73** | IkBe:RelA:p50 🡺IkBe | | 6.00E-05 | constitutive degradation of NFkB within NFkB:IkB complex | min^-1^ | | cytoplasm & nucleus | | | fitted by decreasing the published parameter value[^1^](#_ENREF_1) by 4 fold |  |
| **74** | IkBd:RelA:p50 🡺IkBd | | 6.00E-05 | constitutive degradation of NFkB within NFkB:IkB complex | min^-1^ | | cytoplasm & nucleus | | | fitted by decreasing the published parameter value[^1^](#_ENREF_1) by 4 fold |  |
| **75** | IkBa:RelA:p52 🡺IkBa | | 6.00E-05 | constitutive degradation of NFkB within NFkB:IkB complex | min^-1^ | | cytoplasm & nucleus | | | assumed to be similar to that of RelA:p50 dimer (71). |  |
| **76** | IkBb:RelA:p52 🡺IkBb | | 6.00E-05 | constitutive degradation of  NFkB within NFkB:IkB complex | min^-1^ | | cytoplasm & nucleus | | | assumed to be similar to that of RelA:p50 dimer (72). |  |
| **77** | IkBe:RelA:p52 🡺IkBe | | 6.00E-05 | constitutive degradation of  NFkB within NFkB:IkB complex | min^-1^ | | cytoplasm & nucleus | | | assumed to be similar to that of RelA:p50 dimer (73). |  |
| **78** | IkBd:RelA:p52 🡺IkBd | | 6.00E-05 | constitutive degradation of  NFkB within NFkB:IkB complex | min^-1^ | | cytoplasm & nucleus | | | assumed to be similar to that of RelA:p50 dimer (74). |  |
| **79** | NEMO-IKK+  IkBa:RelA:p50  🡺RelA:p50 | | 1.95E-03 | NEMO mediated degradation of IkB  in composite species | nM^-1^  min^-1^ | | cytoplasm | | | refined the published parameter value[^1^](#_ENREF_1) with a 1.4 fold increase based on experimental measurements (appendix figure 3B) |  |
| **80** | NEMO-IKK+  IkBb:RelA:p50  🡺RelA:p50 | | 5.00E-04 | NEMO mediated degradation of IkB  in composite species | nM^-1^  min^-1^ | | cytoplasm | | | similarly refined the published parameter value[^1^](#_ENREF_1) with a 1.1 fold increase for fitting |  |
| **81** | NEMO-IKK+  IkBe:RelA:p50  🡺RelA:p50 | | 5.00E-04 | NEMO mediated degradation of IkB  in composite species | nM^-1^  min^-1^ | | cytoplasm | | | similarly refined the published parameter value[^1^](#_ENREF_1) with a 1.8 fold decrease for fitting |  |
| **82** | NEMO-IKK+  IkBa:RelA:p52  🡺RelA:p52 | | 1.95E-03 | NEMO mediated degradation of IkB  in composite species | nM^-1^  min^-1^ | | cytoplasm | | | assumed to be similar to that of RelA:p50 dimer (79) based on experimental data (appendix figure 4A). |  |
| **83** | NEMO-IKK+  IkBb:RelA:p52  🡺RelA:p52 | | 5.00E-04 | NEMO mediated degradation of IkB  in composite species | nM^-1^  min^-1^ | | cytoplasm | | | similarly assumed to be similar to that of RelA:p50 dimer (80). |  |
| **84** | NEMO-IKK+  IkBe:RelA:p52  🡺RelA:p52 | | 5.00E-04 | NEMO mediated degradation of IkB  in composite species | nM^-1^  min^-1^ | | cytoplasm | | | similarly assumed to be similar to that of RelA:p50 dimer (81). |  |
| **85** | NIK-IKK1 +IkBd:RelA:p50 🡺RelA:p50 | | 1.00E-03 | NIK mediated degradation of IkB in composite species | nM^-1^  min^-1^ | | cytoplasm | | | refined the published parameter value[^2^](#_ENREF_2) with a 1.2 fold decrease based on experimental measurements (appendix figure 3C) suggesting similar degradation of bound and free IκBδ. |  |
| **86** | NIK-IKK1 +IkBd:RelA:p52  🡺 RelA:p52 | | 1.00E-03 | NIK mediated degradation of IkB  in composite species | nM^-1^  min^-1^ | | cytoplasm | | | assumed to be similar to that of RelA:p50 dimer (85). |  |
| **87** | IkBa:RelA:p50  🡺 IkBa:RelA:p50n | | 2.70E-01 | nuclear import of composite species | min^-1^ | | ___ | | | identical as in[^2^](#_ENREF_2) |  |
| **88** | IkBb:RelA:p50  🡺 IkBb:RelA:p50n | | 2.70E-02 | nuclear import of composite species | min^-1^ | | ___ | | | identical as in[^2^](#_ENREF_2) |  |
| **89** | IkBe:RelA:p50  🡺 IkBe:RelA:p50n | | 1.30E-01 | nuclear import of composite species | min^-1^ | | ___ | | | identical as in[^2^](#_ENREF_2) |  |
| **90** | IkBd:RelA:p50  🡺 IkBd:RelA:p50n | | 2.70E-01 | nuclear import of composite species | min^-1^ | | ___ | | | identical as in[^2^](#_ENREF_2) |  |
| **91** | IkBa:RelA:p52  🡺 IkBa:RelA:p52n | | 2.70E-01 | nuclear import of composite species | min^-1^ | | ___ | | | assumed to be similar to that of RelA:p50 dimer (87). Note, nuclear import of NF-κB:IκB complexes are determined by the nuclear localization signal present in RelA. |  |
| **92** | IkBb:RelA:p52  🡺 IkBb:RelA:p52n | | 2.70E-02 | nuclear import of composite species | min^-1^ | | ___ | | | similarly assumed to be similar to that of RelA:p50 dimer (88). |  |
| **93** | IkBe:RelA:p52  🡺 IkBe:RelA:p52n | | 1.30E-01 | nuclear import of composite species | min^-1^ | | ___ | | | similarly assumed to be similar to that of RelA:p50 dimer (89). |  |
| **94** | IkBd:RelA:p52  🡺 IkBd:RelA:p52n | | 2.70E-01 | nuclear import of composite species | min^-1^ | | ___ | | | similarly assumed to be similar to that of RelA:p50 dimer (90). |  |
| **95** | IkBa:RelA:p50n  🡺 IkBa:RelA:p50 | | 8.30E-01 | nuclear export of composite species | min^-1^ | | ___ | | | identical as in[^2^](#_ENREF_2) |  |
| **96** | IkBb:RelA:p50n  🡺 IkBb:RelA:p50 | | 4.10E-01 | nuclear export of composite species | min^-1^ | | ___ | | | identical as in[^2^](#_ENREF_2) |  |
| **97** | IkBe:RelA:p50n  🡺 IkBe:RelA:p50 | | 4.10E-01 | nuclear export of composite species | min^-1^ | | ___ | | | identical as in[^2^](#_ENREF_2) |  |
| **98** | IkBd:RelA:p50n  🡺 IkBd:RelA:p50 | | 4.10E-01 | nuclear export of composite species | min^-1^ | | ___ | | | identical as in[^2^](#_ENREF_2) |  |
| **99** | IkBa:RelA:p52n  🡺 IkBa:RelA:p52 | | 8.30E-01 | nuclear export of composite species | min^-1^ | | ___ | | | assumed to be similar to that of RelA:p50 dimer (95). Note, nuclear exports of NF-κB:IκB complexes are largely controlled through IκB-derived nuclear export signal. |  |
| **100** | IkBb:RelA:p52n  🡺 IkBb:RelA:p52 | | 4.10E-01 | nuclear export of composite species | min^-1^ | | ___ | | | similarly assumed to be similar to that of RelA:p50 dimer (96). |  |
| **101** | IkBe:RelA:p52n 🡺 IkBe:RelA:p52 | | 4.10E-01 | nuclear export of composite species | min^-1^ | | ___ | | | similarly assumed to be similar to that of RelA:p50 dimer (97). |  |
| **102** | IkBd:RelA:p52n  🡺 IkB2:RelA:p52 | | 4.10E-01 | nuclear export of composite species | min^-1^ | | ___ | | | similarly assumed to be similar to that of RelA:p50 dimer (98). |  |
| **103** | p100+p100🡺IkBd | | 8.10E-02 | p100 association | nM^-1^  min^-1^ | | cytoplasm | | | association/dissociation rates were kept similar to that of NFκB-IκB association/dissociation rates, those captured the experimentally observed 3-5 fold induction of RelA/NFκB during LTβR signaling (Figure 1 - figure supplement 1). |  |
| **104** | IkBd🡺p100+p100 | | 1.20E-05 | IkBd dissociation min^-1^ | |  | cytoplasm | | same as 103 | | |
| **105** | | NIK-IKK1 +p100🡺NFkB2 | 4.20E-03 | p100 processing nM^-1^  through NIK min^-1^ | | cytoplasm | | fitted based on experimentally observed time kinetics of LTβR stimulated NIK induced IκBδ/p100 degradation (appendix figure 3C) and RelA/NFκB activation (Figure 1 - figure supplement 1). | | | |

As such, out of the total 105-parameter values (Supplementary file-2), 34 were identical to those published in earlier model versions^1,2^. Moreover, 20 were derived from the published literature, but were subjected to a minor < 3 fold modification for adapting to the NFκB Systems Model version 1.0. Another 4 parameters were modified < 5 fold for fitting. For additional 12 parameters, further experimental evidences were provided to justify the alterations. Furthermore, another 27 parameters related to the newly described RelA:p52 dimer (Supplementary file-2 and Supplementary file-3) were assumed to be identical to those of RelA:p50 dimer and the assumption was justified using our own experimental measurements and literature. An exception was being made for the association rates underlying RelA:p52-IκB complex formation (a total of 8 parameters) basing on our experimental analyses.

**Related References:**

1 Shih, V. F. *et al.* Control of RelB during dendritic cell activation integrates canonical and noncanonical NF-kappaB pathways. *Nature immunology* **13**, 1162-1170, doi:10.1038/ni.2446 (2012).

2 Basak, S. *et al.* A fourth IkappaB protein within the NF-kappaB signaling module. *Cell* **128**, 369-381, doi:10.1016/j.cell.2006.12.033 (2007).

3 Shih, V. F. *et al.* Kinetic control of negative feedback regulators of NF-kappaB/RelA determines their pathogen- and cytokine-receptor signaling specificity. *Proceedings of the National Academy of Sciences of the United States of America* **106**, 9619-9624, doi:0812367106 [pii] 10.1073/pnas.0812367106 (2009).

4 Hebert, D. N. & Molinari, M. In and out of the ER: protein folding, quality control, degradation, and related human diseases. *Physiol Rev* **87**, 1377-1408, doi:10.1152/physrev.00050.2006 (2007).
