# Supplementary material for: Stimulus-selective crosstalk via the NF-κB signaling system reinforces innate immune response to alleviate gut infection
Source: eLife. 2015 Apr 23;4:e05648. doi: 10.7554/eLife.05648 (PMC4432492; doi:10.7554/eLife.05648)
Supplement: Supplementary file 3. — List of newly described biochemical parameters in the Systems Model v1.0. DOI: http://dx.doi.org/10.7554/eLife.05648.022 [file elife05648s005.docx]

**Supplementary file 3.** **List of newly described biochemical parameters in the Systems Model *v*1.0**

| **Biochemical reaction** | **Reaction no. as in Table 2** | **Corresponding modeled reaction** | **Justification for the parameter value** |
| --- | --- | --- | --- |
| constitutive transcription of NFkB1 mRNA | 5 | 🡺tNFkB1 | tNFkB1 is a composite species encoding RelA:p50 dimer. The reaction rate is fitted to obtain a peak values of ~150nM RelA:p50 dimer at 30min during canonical TNF signaling, as also modeled earlier (Basak et al. al., 2007). |
| inducible transcription of IκBs and p100/*Nfkb2* by RelA:p52 dimer | 10 | 🡺tIkBa | Assumed to be identical with that of RelA:p50. This assumption is further supported by our experimental data presented in appendix figure 4B and 4C. |
|  | 11 | 🡺tIkBb |  |
|  | 12 | 🡺tIkBe |  |
|  | 13 | 🡺tp100 |  |
| degradation rate of NFkB1 mRNA | 18 | tNFkB1 🡺 | Assumed to be similar to that of p100/*Nfkb2* mRNA degradation rates. Along with the constitutive synthesis rate of tNFkB1, this degradation rate provides for nuclear induction of ~150nM RelA:p50 dimer during TNF signaling. |
| constitutive degradation of RelA:p52 | 30 | RelA:p52 🡺 | Assumed to be identical with that of RelA:p50. |
| nuclear import export rates of RelA:p52 dimer | 40 | RelA:p52 🡺 RelA:p52n | Assumed to be identical with RelA:p50. Note, nuclear import/exports are largely determined by the nuclear localization sequence present in RelA. |
|  | 46 | RelA:p52n 🡺 RelA:p52 |  |
| association and dissociation rates between NFkB and IkB | 51 | RelA:p52 + IkBa 🡺IkBa:RelA:p52 | Relative to RelA:p50 dimer, RelA:p52 binding to IκBs were weak as demonstrated in Appendix-1, appendix figure 4D. Accordingly, slower association rates for RelA:p52 binding to IκBs were used to reflect the dissimilar binding affinities. However, dissociation rates were assumed to be identical with that of RelA:p50-IκB. |
|  | 52 | RelA:p52 + IkBb 🡺IkBb:RelA:p52 |  |
|  | 53 | RelA:p52 + IkBe 🡺IkBe:RelA:p52 |  |
|  | 54 | RelA:p52 + IkBd 🡺IkBd:RelA:p52 |  |
|  | 59 | IkBa:RelA:p52 🡺RelA:p52 + IkBa |  |
|  | 60 | IkBb:RelA:p52 🡺RelA:p52 + IkBb |  |
|  | 61 | IkBe:RelA:p52 🡺RelA:p52 + IkBe |  |
|  | 62 | IkBd:RelA:p52 🡺RelA:p52 + IkBd |  |
| constitutive degradation of IkBs and NFkB dimers within the NFkB:IkB complexes | 67 | RelA:p52:IkBa 🡺RelA:p52 | Assumed to be identical with that of the respective RelA:p50-IκB complexes. |
|  | 68 | RelA:p52:IkBb 🡺RelA:p52 |  |
|  | 69 | RelA:p52:IkBe 🡺RelA:p52 |  |
|  | 70 | RelA:p52:IkBd 🡺RelA:p52 |  |
|  | 75 | RelA:p52:IkBa 🡺IkBa |  |
|  | 76 | RelA:p52:IkBb 🡺IkBb |  |
|  | 77 | RelA:p52:IkBe 🡺IkBe |  |
|  | 78 | RelA:p52:IkBd 🡺IkBd |  |
| kinase mediated degradation of IkBs bound to RelA:p52:IkB complex | 83 | NEMO+ IkBa:RelA:p52 🡺RelA:p52 | Assumed to be identical with those of RelA:p50 dimer. Our assumption is supported by the experimental data in Appendix-1, appendix figure 4A. |
|  | 84 | NEMO+ IkBb:RelA:p52 🡺RelA:p52 |  |
|  | 85 | NEMO+ IkBe:RelA:p52 🡺RelA:p52 |  |
| nuclear Import Export rates of NFkB:IkB | 91 | IkBa:RelA:p52🡺 IkBa:RelA:p52n | Assumed to be identical with RelA:p50:IκB molecules. Note, nuclear import of NF-κB:IκB complexes are largely determined by the nuclear localization signal present in RelA, while nuclear export is controlled through IκB-derived nuclear export signal. |
|  | 92 | IkBb:RelA:p52🡺 IkBb:RelA:p52n |  |
|  | 93 | IkBe:RelA:p52🡺 IkBe:RelA:p52n |  |
|  | 94 | IkBd:RelA:p52🡺 IkBd:RelA:p52n |  |
|  | 99 | IkBa:RelA:p52n🡺 IkBa:RelA:p52 |  |
|  | 100 | IkBb:RelA:p52n🡺 IkBb:RelA:p52 |  |
|  | 101 | IkBe:RelA:p52n🡺 IkBe:RelA:p52 |  |
|  | 102 | IkBd:RelA:p52n🡺 IkBd:RelA:p52 |  |
| Association of p100 and dissociation of IkBd | 103 | p100+p100🡺IkBd | association/dissociation rates were kept similar to that of NF-κB-IκB association/dissociation rates those captured experimentally observed 3-5 fold induction of RelA/NF-κB during LTβR signaling (compare appendix figure 5 and Figure-1 Figure Supplement 1). |
|  | 104 | IkBd🡺p100+p100 |  |
| NIK-IKK1 mediated processing of p100 | 105 | NIK+p100🡺NFkB2 | fitted based on experimentally observed time kinetics of LTβR stimulated NIK induced IκBδ/p100 degradation (Appendix-1 appendix figure 3C) and RelA/NF-κB activation (Figure 1 - figure supplement 1). |
